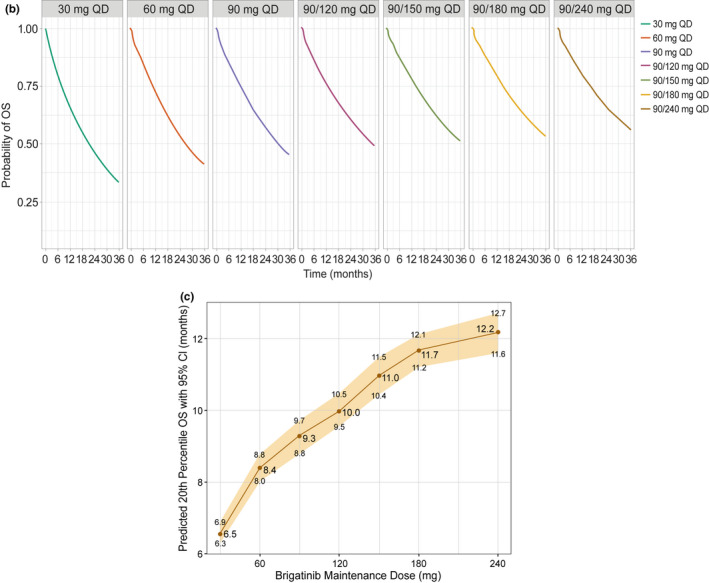# Corrigendum to: Brigatinib dose rationale in anaplastic lymphoma kinase–positive non‐small cell lung cancer: Exposure–response analyses of pivotal ALTA study

**DOI:** 10.1002/psp4.12698

**Published:** 2021-08-19

Neeraj Gupta, Xiaohui Wang, Elliot Offman, Benjamin Rich, David Kerstein, Michael Hanley, Paul M. Diderichsen, Pingkuan Zhang, Karthik Venkatakrishnan. *CPT Pharmacometrics Syst. Pharmacol*. (2020) 9, 718–730; https://doi.org/10.1002/psp4.12569

In the published version of the above article, a coding error in the simulation code was discovered that affected the results of the exposure‐response analyses for efficacy endpoints of the progression‐free survival (PFS), intracranial PFS (iPFS), and overall survival (OS) parametric time‐to‐event models. Upon correction of the coding error, the recalculated results were mostly comparable to those in the original publication, which are reflected in the revised figures and text presented hereafter. The predicted median PFS and iPFS for the 90 and 180 mg every day (q.d.) regimens were slightly shorter than previously calculated. Consequently, the model‐predicted probabilities of OS for these regimens were lower than previously calculated, resulting in probabilities that were similar to, rather than higher than, the observed data estimates.

These errors do not change the conclusion that a favorable benefit‐risk profile exists for the approved dosing regimen (180 mg q.d. with 7‐day lead‐in at 90 mg) versus 90 mg q.d.

Revisions in the Text are tabulated and shown below:

**Table:** 

Section and paragraph	Where it reads	The sentence should read
Results, paragraph 2, last sentence	For “Simulations from the final model predicted median (95% CI) PFS values at 90 mg q.d. and 180 mg q.d. of 12.0 (11.6–12.3) months and 14.7 (14.2–15.2) months, respectively (Figure 1c,d).”	Read “Simulations from the final model predicted median (95% CI) PFS values at 90 mg q.d. and 180 mg q.d. of 10.2 (9.9–10.4) months and 12.4 (12.0–12.7) months, respectively (Figure 1c,d).”
Results, paragraph 3, last sentence	For “Simulations based on the final TTE model estimated that the predicted median (95% CI) iPFS values at exposures associated with the 90‐mg q.d. and 180‐mg q.d. regimens were 15.1 (14.8–15.5) months and 19.2 (18.7–19.7) months, respectively (Figure 2b,c).”	Read “Simulations based on the final TTE model estimated that the predicted median (95% CI) iPFS values at exposures associated with the 90‐mg q.d. and 180‐mg q.d. regimens were 12.7 (12.4–13.1) months and 15.7 (15.3–16.2) months, respectively (Figure 2b,c).”
Results, paragraph 4, last sentence	For “Simulations predicted that the 20th percentile (95% CI) of OS (i.e., the time at which 80% of patients are expected to remain alive) was 12.4 (12.0–13.0) months with the 90 mg q.d. dosing regimen and 15.8 (15.2–16.5) months with the 180 mg q.d. regimen (Figure 3b,c).”	Read “Simulations predicted that the 20th percentile (95% CI) of OS (i.e., the time at which 80% of patients are expected to remain alive) was 9.3 (8.8–9.7) months with the 90‐mg q.d. dosing regimen and 11.7 (11.2–12.1) months with the 180‐mg q.d. regimen (Figure 3b,c).”
Discussion, paragraph 4	For “The model‐predicted median PFS values of 12.0 months for 90 mg q.d. and 14.7 months for 180 mg q.d. …”	Read “The model‐predicted median PFS values of 10.2 months for 90 mg q.d. and 12.4 months for 180 mg q.d. …”
Discussion, paragraph 4	For “The predicted advantage for the 240 mg dose compared with the 180 mg dose for median PFS was 16.0 vs. 14.7 months, for median iPFS was 21.4 vs. 19.2 months, and for the time to 20th percentile OS was 17.3 vs. 15.8 months.”	Read “The predicted advantage for the 240‐mg dose compared with the 180‐mg dose for median PFS was 13.3 vs 12.4 months, for median iPFS was 16.9 vs 15.7 months, and for the time to 20th percentile OS was 12.2 vs 11.7 months.”
Discussion, paragraph 5	For “Predicted median iPFS was 15.1 and 19.2 months for the 90 and 180 mg q.d. doses, respectively…”	Read “Predicted median iPFS was 12.7 and 15.7 months for the 90‐ and 180‐mg q.d. doses, respectively…”
Discussion, paragraph 6	For “For the 90 mg and 180 mg regimens, the predicted times at which 80% of patients would remain alive were 12.4 and 15.8 months, respectively. Increasing the daily dose to 240 mg per day is predicted to result in 80% of patients surviving at 17.3 months.”	Read “For the 90‐mg and 180‐mg regimens, the predicted times at which 80% of patients would remain alive were 9.3 and 11.7 months, respectively. Increasing the daily dose to 240 mg per day is predicted to result in 80% of patients surviving at 12.2 months.”

The revised figures are shown below:

Revised Figure 1c,d:

**Figure 1:**
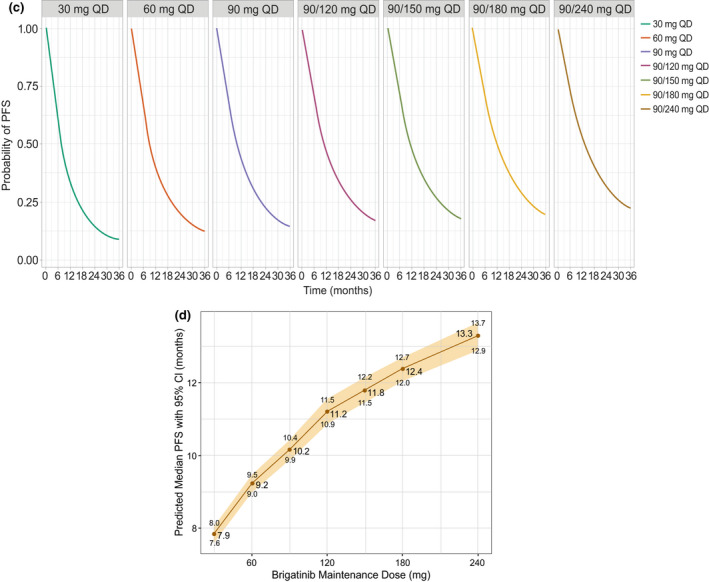


Revised Figure 2b,c:

**Figure 2:**
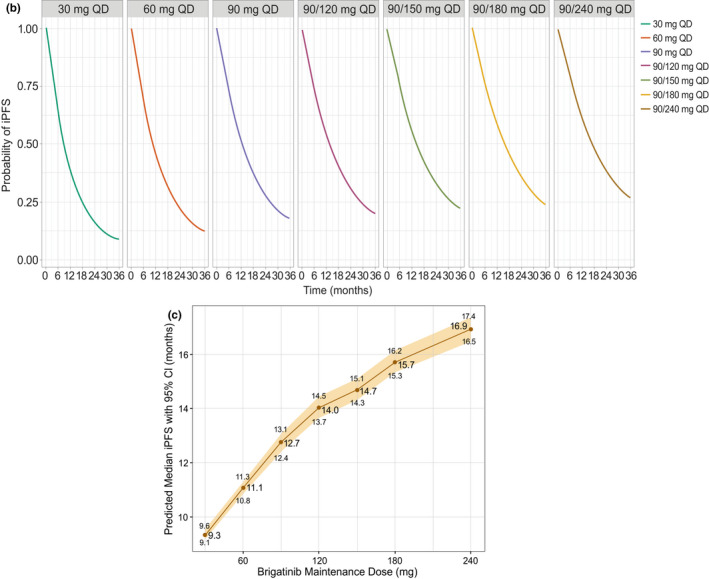


Revised Figure 3b,c:

**Figure 3:**